# Identification of Biomarkers Related to Immune Cell Infiltration with Gene Coexpression Network in Myocardial Infarction

**DOI:** 10.1155/2021/2227067

**Published:** 2021-11-05

**Authors:** Lei Zhang, Qiqi Wang, Xudong Xie

**Affiliations:** ^1^Department of Electrocardiogram, The First Affiliated Hospital, Zhejiang University School of Medicine, Hangzhou, 310003, China; ^2^Department of Cardiology, The First Affiliated Hospital, Zhejiang University School of Medicine, China

## Abstract

**Background:**

There is evidence that the immune system plays a key critical role in the pathogenesis of myocardial infarction (MI). However, the exact mechanisms associated with immunity have not been systematically uncovered.

**Methods:**

This study used the weighted gene coexpression network analysis (WGCNA) and the CIBERSORT algorithm to analyze the MI expression data from the Gene Expression Omnibus database and then identify the module associated with immune cell infiltration. In addition, we built the coexpression network and protein-protein interactions network analysis to identify the hub genes. Furthermore, the relationship between hub genes and NK cell resting was validated by using another dataset GSE123342. Finally, receiver operating characteristic (ROC) curve analyses were used to assess the diagnostic value of verified hub genes.

**Results:**

Monocytes and neutrophils were markedly increased, and T cell CD8, T cell CD4 naive, T cell CD4 memory resting, and NK cell resting were significantly decreased in MI groups compared with stable coronary artery disease (CAD) groups. The WGCNA results showed that the pink model had the highest correlation with the NK cell resting infiltration level. We identified 11 hub genes whose expression correlated to the NK cell resting infiltration level, among which, 7 hub genes (NKG7, TBX21, PRF1, CD247, KLRD1, FASLG, and EOMES) were successfully validated in GSE123342. And these 7 genes had diagnostic value to distinguish MI and stable CAD.

**Conclusions:**

NKG7, TBX21, PRF1, CD247, KLRD1, FASLG, and EOMES may be a diagnostic biomarker and therapeutic target associated with NK cell resting infiltration in MI.

## 1. Introduction

Myocardial infarction (MI) is the most severe manifestations cardiac event and remains the leading cause of mortality worldwide [1]. It can lead to loss of cardiomyocytes, left ventricular remodeling, decreased cardiac function, and potentially heart failure. MI is associated with a variety of factors, including gender, age, smoking, hypertension, and diabetes complications [2]. Microarray analysis is a widely used strategy for detecting novel biomarkers for diagnosis, prediction of disease severity, and identification of new drug targets [3]. At present, many studies have identified biomarkers that distinguish MI from normal controls based on the microarray analysis and RNA sequencing. For instance, several circulating genes (MAX, BCL3, NCOA7, CCL5, GTF3C2, etc.) are potential biomarkers that distinguish MI from normal controls and may play important roles in MI development [4]. Zhao et al. find that eight genes, IFIT3, MX1, HLA-DQA1, RORA, PTGDS, CRIP2, COL6A2, and S100P, may be considered biomarkers between MI and normal controls. Coronary artery disease (CAD) is the main type of cardiovascular disease, which causes heavy economic and social burden worldwide [5]. MI is one of the most severe manifestations of stable CAD and the leading cause of death from noninfectious diseases worldwide. As far as we know, few studies have explored biomarkers that distinguish MI from stable CAD. Thence, it is an urgent need for new biomarkers with high sensitivity and specificity to achieve early diagnosis between MI and stable CAD.

Infiltrating cells exhibit specific spatial and temporal distribution and activity patterns, while simultaneously engaging in active, continuous cross talk with each other and with other cardiac cells cardiomyocytes [6]. This forms a highly complex regulatory pattern, which plays a crucial role in the normal healing of the heart after MI [7, 8]. Inflammatory processes can also lead to hypertrophy, fibrosis, and other types of heart damage which can then cause heart failure [9]. MI causes aseptic inflammation, manifested as recruitment and activation of innate and adaptive immune system cells [6]. Therefore, immunoregulatory therapy has great potential in accelerating cardiac repair and improving left ventricular remodeling after MI. In order to find the optimal immunoregulatory therapy, it is necessary to uncover the temporal dynamics of immune cell accumulation after MI. In previous studies, immunohistochemical methods used to examine immune cells relied on a single marker to identify a specific subset of immune cells but acquired poor immunohistochemical results when a small number of cells or cell types were detected may be misleading [10–12]. Therefore, a comprehensive understanding of the immune response between MI and stable CAD is necessary.

With the wide application and continuous development of bioinformatics technology, many algorithms have been developed to discover novel biomarkers [13]. Weighted gene coexpression network analysis (WGCNA) is a system biology method that is widely used for building coexpression gene modules and searching for biomarkers at the transcription level [14, 15]. Lately, a novel deconvolution algorithm, known as Cell-type Identification by Estimating Relative Subsets of RNA Transcripts (CIBERSORT), is established and used to approximate the cellular composition of immune cells. This analytical tool has been applied to quantify the level of immune cell infiltration in several cancers like prostate cancer, colorectal cancer, gastric cancer, and renal cell carcinoma [12, 16–18].

Currently, it is an urgent problem to find diagnostic biomarkers between MI and stable CAD and to comprehensively understand the immune response between MI and CAD. The goal of this study was to reveal immune-related biomarkers for the diagnosis of MI and stable CAD through gene expression data from Gene Expression Omnibus (GEO) datasets. To explore the role of immune cells and identify potential biomarkers MI and stable CAD, we used WGCNA to process gene expression data of MI and stable CAD. Then, immune cell infiltration of samples was calculated applying the CIBERSORT. In addition, we identified key modules and hub genes associated with the level of NK cell resting infiltration and finally validated the immunological and clinical characteristics of these genes through database analysis. To our knowledge, this is the first time that WGCNA has been used to identify NK cell resting-related biomarkers in MI. This study provides a theoretical basis for finding biomarkers for early diagnosis and immunotherapy targets of MI and CAD in the future.

## 2. Results

### 2.1. Data Preprocessing

**Figure 1:**
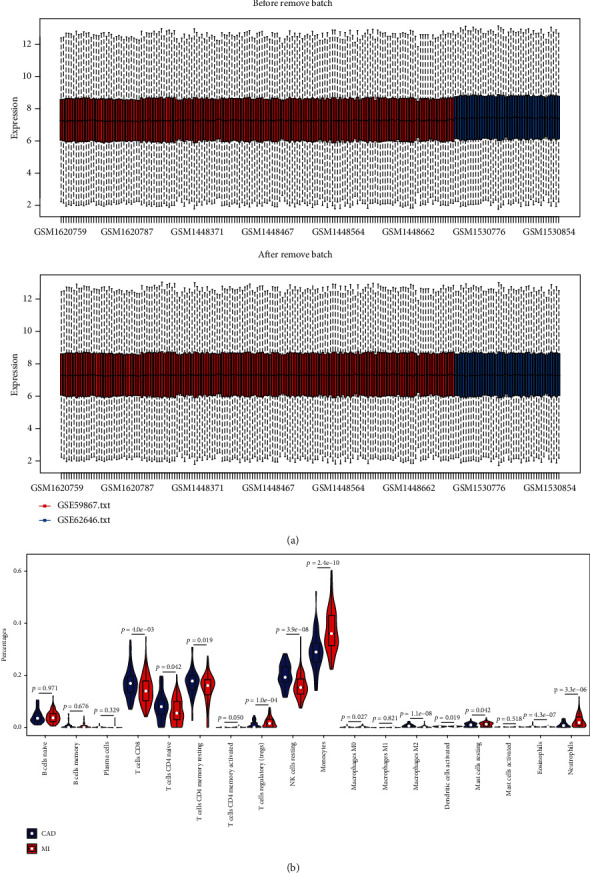
(a) The expression matrix from 199 samples in the training dataset. (b) The landscape of tumor-infiltrating immune cells. The difference of the proportions of tumor-infiltrating immune cells between MI and stable CAD sample.

The expression matrix of 2 GEO datasets (GSE59867 and GSE62646) containing 139 MI patients and 60 CAD control samples was downloaded. Samples of 2 GEO datasets were combined into a training dataset containing 18,837 genes. After batch effect correction with ComBat, we obtained the expression matrix from 199 samples in the training dataset (Figure 1(a)).

### 2.2. Evaluation of Immune Cell Infiltration

The differential of immune cell infiltration between MI and stable CAD groups was evaluated by the CIBERSOFT algorithm. The distributions of immune infiltration cells in two groups are displayed in Figure 1(b). We found that monocytes, neutrophils, T cell CD8, T cell CD4 naive, T cell CD4 memory resting, and NK cell resting were obviously altered between MI and stable CAD groups, while B cell naive, B cell memory, plasma cells, T cell CD4 memory activated, T cell regulatory (Tregs), macrophage M0, macrophage M1, macrophage M2, dendritic cell activated, mast cell resting, mast cell activated, and eosinophils were not significantly changed between groups. Among them, monocytes and neutrophils were markedly increased and T cell CD8, T cell CD4 naive, T cell CD4 memory resting, and NK cell resting were significantly decreased in MI groups compared with stable CAD groups. These results indicate that the occurrence and development of MI may be closely related to immune cells.

### 2.3. Weighted Gene Coexpression Network Construction and Hub Module Identification

**Figure 2:**
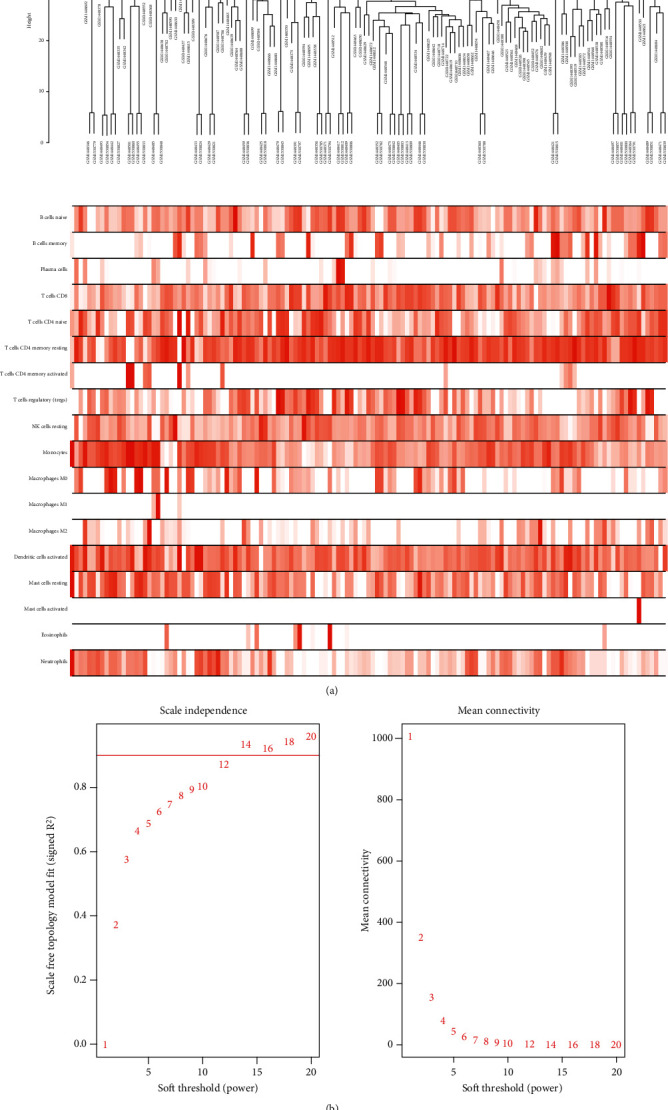
WGCNA revealed gene coexpression networks. (a) Clustering dendrogram of 139 samples corresponding to clinical characteristics. (b) Analysis of the scale-free fit index and mean connectivity for various soft-thresholding powers (*β*).

**Figure 3:**
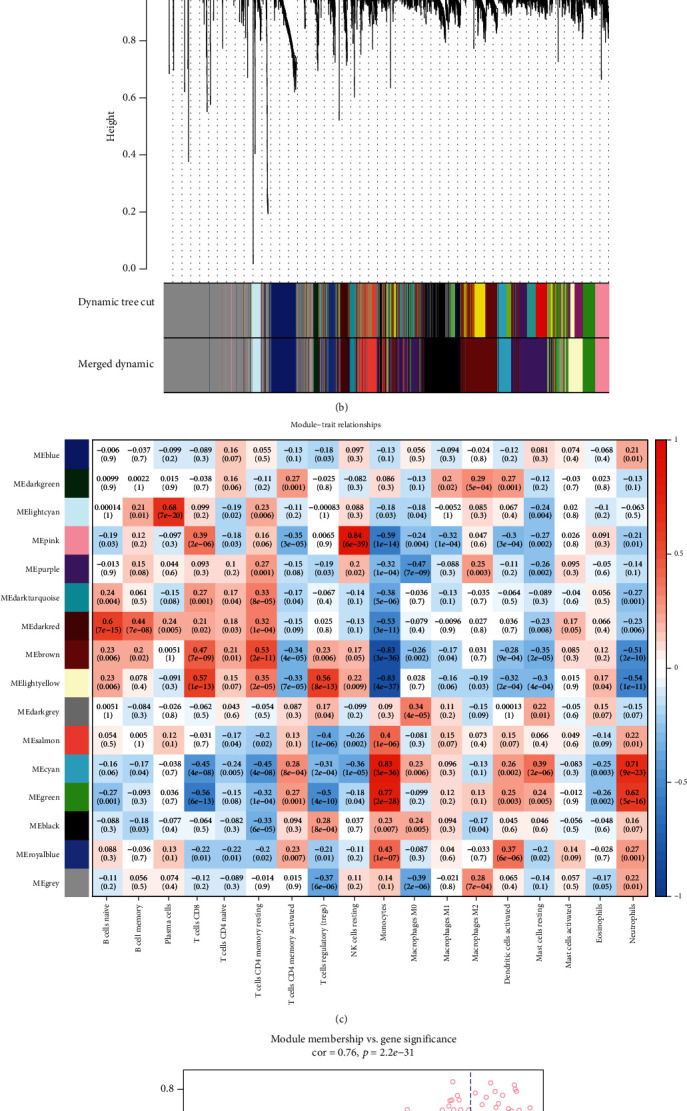
Identification of gene modules associated with the immune cell infiltration of MI. (a) The horizontal line defines the threshold, so 15 distinct genes modules were identified. (b) The dendrogram of all genes is clustered based on a dissimilarity measure. (c) The heatmap shows the correlation between MEs and the immune cell infiltration of MI. (d) The scatter plot shows the correlation between gene significance for MI and module membership in pink module.

**Figure 4:**
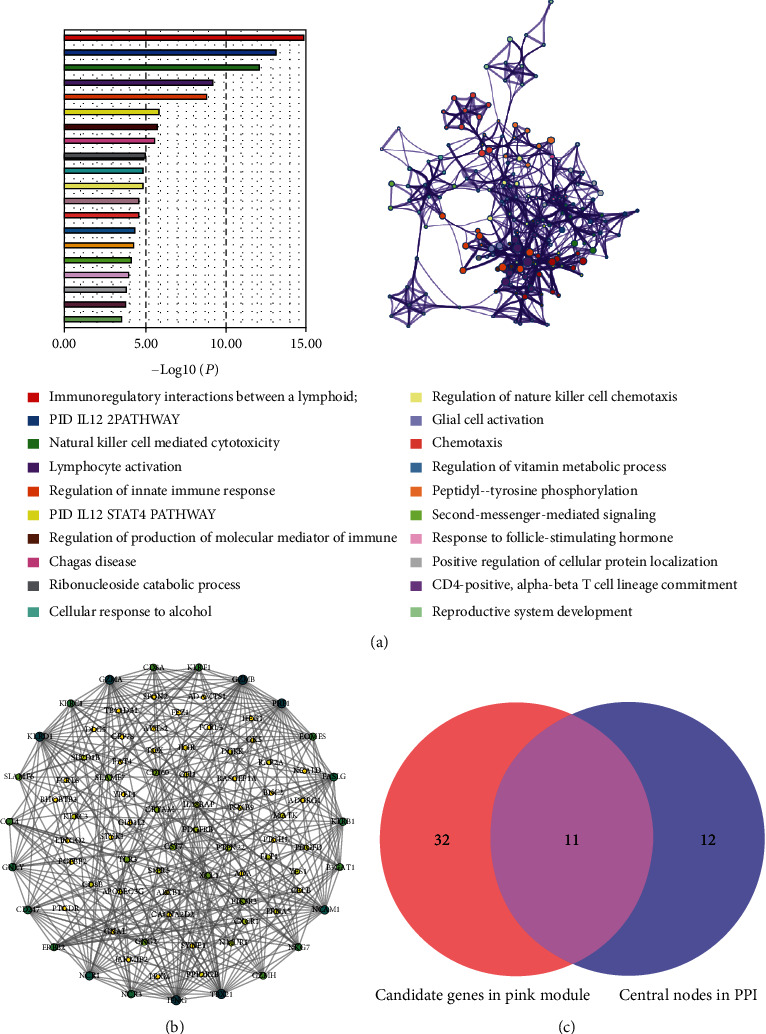
Key modules and identification of hub genes. (a) The first 20 enriched terms are shown as a bar chart on the left. The network diagram on the right is constructed with each enrichment term as a node and the similarity of the node as the edge. Nodes with the same cluster ID are the same color. (b) PPI network of genes from the pink module. The higher the number of connected nodes, the larger the size of the node. (c) Hub genes were selected based on overlap between PPI and coexpression networks.

To explore the relationship between functional modules and immune cell infiltration in patients with MI, we selected the top 25% variance genes, including 4709 genes for WGCNA. The sample dendrogram and trait heatmap of 139 samples in this study are presented in Figure 2(a). To construct a scale-free network, the power of *β* = 12 was selected the soft-thresholding cut-off standard power (Figure 2(b)). The dynamic tree cutting method was used to merge the modules with dissimilarity of <25%, and finally, 15 modules were determined (Figures 3(a) and 3(b)). Correlation analysis was performed between the eigengenes of each module and immune cell (Figure 3(c)). Compared with other modules, lightcyan module was highly associated with plasma cells infiltration level. (*R*^2^ = 0.68, *P* = 7*E* − 20), pink module was significantly correlated with NK cell resting infiltration level. (*R*^2^ = 0.84, *P* = 6*E* − 39), green module was highly correlated with monocyte infiltration level (*R*^2^ = 0.77, *P* = 2*E* − 28), and cyan module was related to monocyte (*R*^2^ = 0.83, *P* = 3*E* − 36) and neutrophil (*R*^2^ = 0.71, *P* = 9*E* − 23) infiltration levels, respectively. Among which, the pink model state had the highest correlation with the NK cell resting infiltration level. We therefore selected the pink model for subsequent analysis. We performed an intramodular analysis for the pink module, and the module membership and gene significance showed a very meaningful correlation (cor = 0.76 and *P* value = 2.2*E* − 31), indicating that the 160 genes in the pink module tend to be remarkably correlated with NK cells resting infiltration level (Figure 3(d)). Thence, the pink module was identified as the hub module associated with MI. To uncover the affected functions of the genes clustered in the pink module, we carried out the GO and KEGG pathways using the Metascape tool. The 20 highest enrichment terms were all immune-related terms and are presented in Figure 4(a), and the three most highly enriched terms were immunoregulatory interactions between a lymphoid and a nonlymphoid cell, PID IL12 2PATHWAY, and natural killer cell-mediated cytotoxicity.

### 2.4. Identification and Validation of Hub Genes

**Figure 5:**
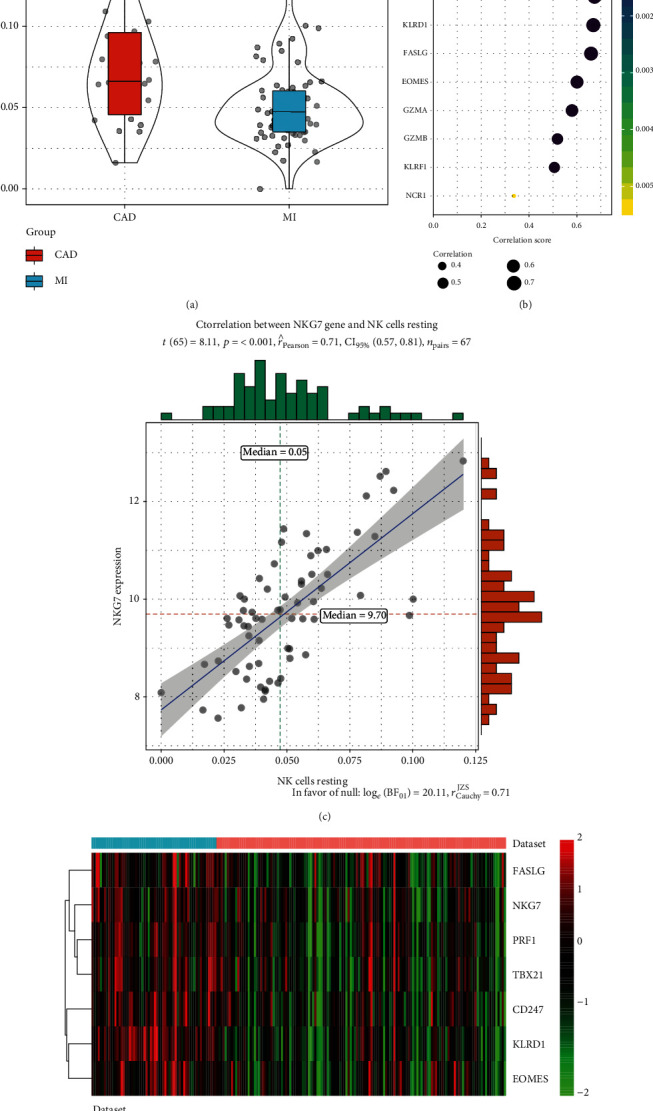
Validation of hub genes. (a) NK cell resting infiltration level between MI and stable CAD. (b) Relationship between 11 hub genes expression and NK cell resting infiltration level. *P* < 0.05 is considered statistically significant. (c) Scatter plot of NKG7 expression and NK cell resting infiltration level. (d) Hierarchical clustering analysis of 7 verified genes.

The genes that were highly linked to the pink module associated with the level of NK cell resting infiltration were studied. According to the cut-off threshold (module membership > 0.8 and gene significance > 0.6), a total of 43 genes were defined as candidate hub genes (Figure 3(d)). Based on the PPI network of the pink module, the gene with degree > 10 was identified to be the central node, and we obtained 23 central nodes and visualized these results using Cytoscape (Figure 4(b)). A total of 11 genes were selected in both analyses designated as hub genes (Figure 4(c); Table 1). To validate the relationship between these 11 hub genes and NK cell resting, we downloaded GSE123342 to analyze the level of NK cell resting infiltration, and the results indicated that the NK cell resting infiltration level was significantly reduced between the MI and stable CAD groups, which is consistent with our previous analysis (Figure 5(a)). The correlation analysis results displayed a positive correlation of the expression values of the 11 genes with the infiltration levels of NK cell resting (correlation coefficient of >0.6 for all genes except GZMA, GZMB, KLRF1, and NCR1; Figure 5(b)). For example, in Figure 5(c), we exhibited a scatter plot of NKG7 expression and NK cell resting infiltration level. The results verified the identified hub genes as highly correlated with the NK cell resting infiltration level, playing key roles in the development of MI.

### 2.5. Hierarchical Clustering and Receiver Operating Characteristic (ROC) Curve Analyses of Verified Hub Genes

**Figure 6:**
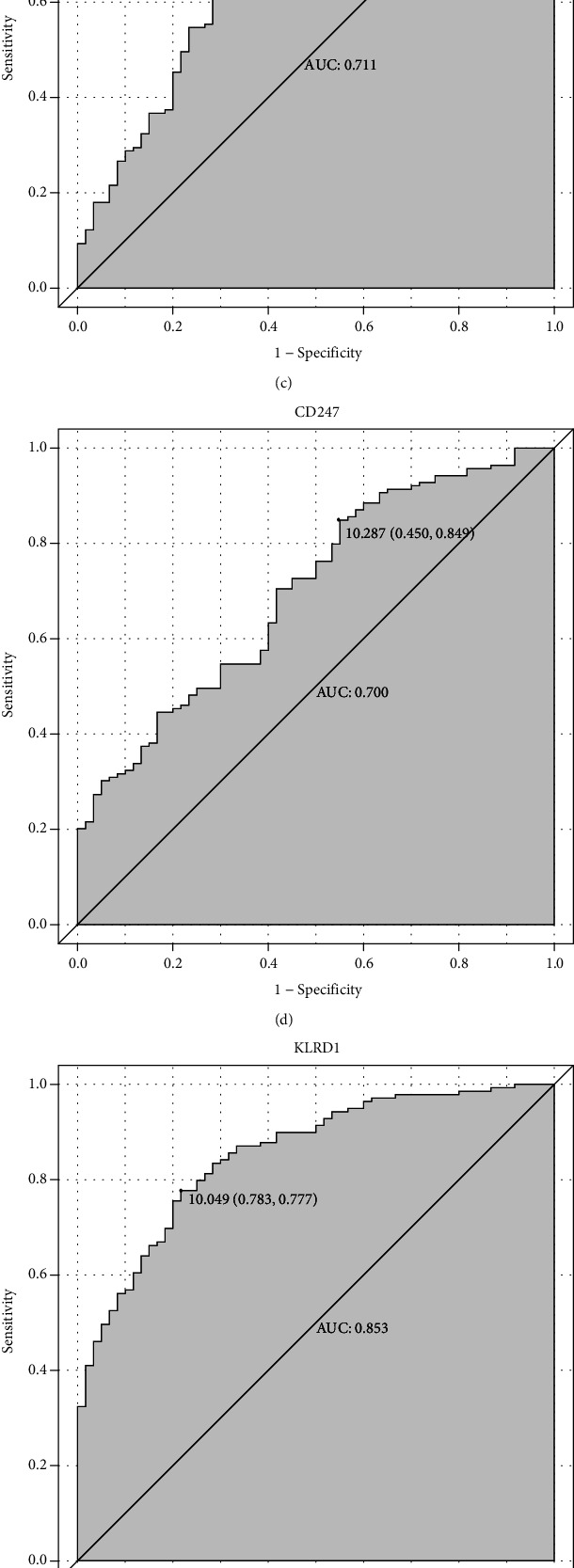
ROC analysis of 7 verified genes. The AUC was analyzed to evaluate the performance of each hub genes. *x*-axis indicated 1-specificity, and *y*-axis indicated sensitivity.

The hierarchical clustering analysis of 7 verified genes (NKG7, TBX21, PRF1, CD247, KLRD1, FASLG, and EOMES) is presented in Figure 5(d). We also evaluated the diagnostic value of NKG7, TBX21, PRF1, CD247, KLRD1, FASLG, and EOMES in MI. The results indicated that NKG7 (AUC = 0.687), TBX21 (AUC = 0.681), PRF1 (AUC = 0.711), CD247 (AUC = 0.700), KLRD1 (AUC = 0.853), FASLG (AUC = 0.658), and EOMES (AUC = 0718) were capable of discriminating MI and stable CAD (Figure 6).

## 3. Discussion

MI is a serious disease with high morbidity and mortality worldwide and affects patient's health and life [19]. During the past years, the number of MI patients increased year by year. Early diagnosis of MI is urgently needed for the effective management of patients and selection of appropriate treatment. Although traditionally available biomarkers have been available to help in the diagnosis of MI, they still lack high specificity. Therefore, it is very urgent to identify new diagnostic biomarkers and therapeutic targets with the smallest risk of adverse reactions and greatest sensitivity and specificity.

MI is an inflammatory disease with multifactorial interactions, including immunization, environmental influences, and genetic factors. The search for biomarkers in the serum, saliva, tissues, and peripheral blood released in inflammatory state has attracted the attention of researchers. For example, the expression of galectin-3 in serum and saliva in patients with periodontitis and periodontitis + coronary heart disease is significantly higher than that in patients with coronary heart disease and healthy controls, indicating that galectin-3 in the serum and saliva may be used as a marker for predicting periodontitis and periodontitis and coronary heart disease [20]. The concentrations of NLRP3 in serum and saliva of patients with periodontitis and periodontitis + type II diabetes mellitus were higher than those of healthy controls and type II diabetes mellitus patients [21]. The concentrations of IL-6 in the saliva of periodontitis patients were significantly higher than that of healthy subjects, and the level of IL-6 in saliva was related to the clinical parameters of periodontitis patients [22]. Th2 cell markers in the peripheral blood have been reported to be sensitive measures of exacerbation of symptoms in patients with asthma and may be used as a biomarker for asthma exacerbation [23]. Since MI is a key cause of myocardial cell injury, cardiac troponin has become one of the important factors of MI diagnosis [24]. Detection of cardiac troponin in peripheral blood suggests myocardial cell injury. At present, peripheral blood biomarkers have been reported as biomarkers for the diagnosis of MI. The combination of microRNA-1291, microRNA-217, microRNA-455-3p, and microRNA-566 in peripheral blood of patients with MI may serve as a new and potential biomarker for the early diagnosis of MI [25]. Circulating microRNA-19a in the peripheral blood is upregulated in the MI patients compared with controls, which may act as a new biomarker for diagnosis of MI [26]. In the current study, we used three datasets of gene expression from peripheral the blood and peripheral monocytes of patients with MI to find immune-related early diagnostic markers between MI and stable CAD.

Here, we identified 11 hub genes whose expression correlated to NK cell resting infiltration level, indicating a potential mechanism through which these genes may be involved in MI development. Of the identified 11 genes, 7 hub genes (NKG7, TBX21, PRF1, CD247, KLRD1, FASLG, and EOMES) were successfully validated in GSE123342. And these 7 genes had a diagnostic value to distinguish MI and stable CAD.

Natural killer cell granule protein 7 (NKG7) is a 17 kDa type III integral membrane protein localized to vesicles containing cytotoxic particles [27]. NKG7 is first found in NK cells and T cell 16, but the molecule has been studied a little so far [28]. A recent study showed that NKG7 is a regulator of lymphocyte granule exocytosis and downstream inflammation in many diseases, and the NKG7 expression of natural killer cells is essential for the control of tumor initiation, progression, and metastasis [29]. Chen et al. have found that NKG7 is downregulated between the MI group and control group [30]. Here, our results indicated that NKG7 expression positively correlated with the NK cell resting infiltration level. In addition, the ROC analysis results showed that NKG7 had a diagnostic value and could distinguish between MI and stable CAD. Thence, we inferred that NKG7 may be considered diagnostic biomarkers for MI.

Perforin (PRF1) belongs to the membrane attack complex/PR (FMACPF) superfamily, a highly conserved glycoprotein that can be secreted by NK cells, CTL cells, and regulatory T cells [30, 31]. Based on its key role in immune monitoring and regulation, PRF1 malfunctions have been reported to be associated with many diseases [32]. A previous study indicated that PRF1 can induce clathrin- and dynein-dependent endocytosis, and suppressing this endocytosis pathway may lead to apoptosis cell death [33]. PRF1 is downregulated in the MI group compared with the control group, and the abnormal expression of PRF1 may be the main reason for the progression of left ventricular dysfunction in MI [30]. The present study found that the PRF1 expression level was positively correlated with the NK cell resting infiltration level, and PRF1 had a diagnostic value and could distinguish between MI and stable CAD. We speculate that PRF1 may be involved in the development of MI, which may be a novel biomarker for the diagnosis of MI. Therefore, further experiments are needed to confirm it.

Fas ligand (FASLG) is a member of the tumor necrosis factor superfamily and major activator of apoptotic pathways binding to tumor necrosis factor receptors during myocardial infarction [34]. Wu et al. have reported that FASLG is downregulated in the MI group compared with the normal group [35]. T-box 21 variant (TBX21) encodes a transcription factor called T-bet, whose primary function is to block Th1 to Th2 cell differentiation [36]. Decreased T-bet changes the drifting Th1/Th2 cells, resulting in the immune imbalance of myocardial tissue after cardiopulmonary resuscitation in a porcine model of cardiac arrest [37]. Killer cell lectin-like receptor D1 (KLRD1), also named CD94, is an antigen preferentially expressed on NK cells. KLRD1 expression is negatively correlated with symptom severity [38]. Chen et al. have reported that TBX21 and KLRD1 are downregulated in the MI group compared with the normal group [30].

Herein, our results displayed that FASLG, TBX21, and KLRD1 expressions positively correlated with the NK cell resting infiltration level. Moreover, FASLG, TBX21, and KLRD1 had a diagnostic value and could distinguish between MI and stable CAD. Therefore, we suspected that FASLG, TBX21, and KLRD1 may be a novel biomarker for the diagnosis of MI.

## 4. Conclusion

In summary, our study is the first time to identify NK cell resting-related biomarkers of MI using the WGCNA and CIBERSORT algorithm. Eleven hub genes were identified which were associated with the level of NK cell resting infiltration. Through the verification of bioinformatics, NKG7, TBX21, PRF1, CD247, KLRD1, FASLG, and EOMES were identified as a potential diagnostic biomarker and target for MI immunotherapy. However, our study has some limitations. Additional sample data is required to validate the present findings, and the specific mechanism of these genes in MI needs further investigation both in vitro and in vivo.

## 5. Materials and Methods

### 5.1. Raw Data Collection

Gene expression profiles of myocardial infarction (MI) were downloaded from the GEO database (http://www.ncbi.nlm.nih.gov/geo) in the National Center of Biotechnology Information (NCBI). This study samples consisted of MI patients and control group patients with stable coronary artery disease (CAD). Three datasets (GSE59867, GSE62646, and GSE123342) were included this study (Table 2). The dataset GSE59867 was collected from in peripheral blood mononuclear cells containing 111 MI patients and 46 stable CAD patients, using the GPL6244 platform of the Affymetrix Human Gene 1.0 ST Array. GSE62646, based on the GPL6244 platform of Affymetrix Human Gene 1.0 ST Array, consisted 14 stable CAD patients and 28 MI patients from peripheral blood mononuclear cells. GSE123342, previously researched using the GPL17586 platform of the Affymetrix Human Transcriptome Array 2.0, included 22 patients and 67 MI patients from the peripheral blood and was used as an independent validation cohort.

### 5.2. Data Preprocessing

The raw data were preprocessed using R language (v.3.6.3). Oligo package in R language was used to normalize the raw data for data processing. The probe level data were converted into corresponding gene expression values. For multiple mapping to a same gene, we applied the average values to represent the expression of that gene. The ComBat function of the R language SVA package was subsequently used to eliminate the heterogeneity between the gene expression data.

### 5.3. Evaluation of Immune Cell Infiltration

Cell-type Identification by Estimating Relative Subsets of RNA Transcripts (CIBERSORT) is an in silico method that has been confirmed by fluorescence-activated cell sorting and can be utilized to evaluate 22 types of immune infiltrating cell composition of bulk samples [39]. CIBERSORT is superior to other algorithms in the identification and elaborate division of immune cells [40]. The gene expression data were uploaded to the CIBERSORT web portal (http://cibersort.stanford.edu/) with the algorithm run using the LM22 signature and 5000 permutations. The Wilcoxon rank-sum test was exploited to compare the differential abundances of immune infiltrates between the MI and stable CAD groups. The distributions of immune cells in two groups were exhibited by the ggplot2 package in R language. Finally, the percentage of immune cells in each sample was selected as the WGCNA trait data.

### 5.4. Weighted Gene Coexpression Network Construction

WGCNA is an approach of clustering genes based on expression patterns, systematically analyzing the relationship between gene modules and traits, and classifying gene functions [41]. We selected the top 25% variance genes, which included 139 MI samples gene expression matrix, to construct a coexpression network using the WGCNA package in R language. First, the expression level of a single transcript was converted into a similarity matrix based on the Pearson correlation value between the paired genes. Next, the similarity matrix was transformed into an adjacency matrix. When the *β* value was 12, the adjacency matrix was then converted to a topological overlap matrix. In order to classify genes with similar expression patterns into different module eigengenes, a dynamic hybrid cutting method was performed, and the minimum module size cut-off value was 30. A hierarchical clustering tree was used to display the result.

### 5.5. Identification of Clinical Significant Modules and Enrichment Analysis

The principal component analysis of all genes was performed in each module and used the value of principal component one as module eigengenes. Then, Pearson's correlation analysis was calculated the correlation between module eigengenes and the infiltration level of T cells and identify the significance of the module. The modules with absolute value of *P* < 0.05 were considered significantly correlated with T cells. In addition, we further calculated and visualized the difference of the module characteristic genes, selected a cutting line for the module tree diagram, and merged some modules. Furthermore, we selected the immune cells of interest and the module with the highest correlation coefficient and identified it as a hub module. To further explore the biological function of genes in the hub module, we used the online tool Metascape (http://metascape.org) to perform Gene Ontology (GO) analysis and the Kyoto Encyclopedia of Genes and Genomics (KEGG) pathway enrichment analysis.

### 5.6. Identification of Hub Genes

We identified candidate hub genes according to the modular connectivity and clinical trait relationship of each gene in the hub module. Module connectivity was measured by the absolute value of the Pearson's correlation (module membership (MM)). Clinical trait relationship is defined as the absolute value of Pearson's correlation between each gene and the trait (gene significance (GS)). The MM > 0.8 and GS > 0.6 were selected as the cut-of criteria for the identification of hub genes. Moreover, all genes in the hub module were selected and used to build the protein-protein interactions (PPI) network using the Search Tool for the Retrieval of Interacting Gene (String) database (https://string-db.org/), and Cytoscape (v 3.7.2) was used to visualize the network. The gene with degree > 10 was considered the central node. We used an online tool (http://www.bioinformatics.com.cn/) to conduct a Venn analysis on the candidate hub genes and the central nodes in the PPI network, and the intersection genes were considered hub genes.

### 5.7. Confirmation of Hub Gene

The GSE123342 dataset was used to verify the correlation between hub gene and immune cells. First, we applied CIBERSORT to assess the content of NK cells in each MI sample. Then, the Spearman correlation between hub gene and NK cells resting immune cells was calculated, and the ggplot2 package (v 3.1.1) in R language was used to visualize the results.

### 5.8. Hierarchical Clustering and Receiver Operating Characteristic (ROC) Curve Analyses of Verified Hub Genes

The hierarchical clustering analysis of the verified hub genes were produced using R language. In order to evaluate the diagnostic value of hub genes, the “pROC” package was performed to generate ROC, and the area under the ROC curve (AUC) represents the diagnostic value. When the AUC value was greater than 0.6, the hub genes were thought to be able to distinguish between MI and stable CAD with good specificity and sensitivity.

## Figures and Tables

**Table 1:** Hub gene in pink module.

Symbol	Module color	GS NK cells resting	*P* (GS NK cells resting)	MM pink	*P* (MM pink)	Degree
GZMB	Pink	0.820247	4.80*E* − 35	0.847659	1.55*E* − 39	27
TBX21	Pink	0.787318	1.45*E* − 30	0.876234	2.83*E* − 45	26
GZMA	Pink	0.607282	2.27*E* − 15	0.84347	8.54*E* − 39	25
KLRD1	Pink	0.747353	4.29*E* − 26	0.91546	5.13*E* − 56	25
PRF1	Pink	0.814962	2.88*E* − 34	0.904279	1.72*E* − 52	25
NCR1	Pink	0.797583	7.14*E* − 32	0.85801	1.81*E* − 41	21
CD247	Pink	0.718785	2.21*E* − 23	0.849544	7.06*E* − 40	19
FASLG	Pink	0.793318	2.54*E* − 31	0.810763	1.15*E* − 33	19
NKG7	Pink	0.765204	5.55*E* − 28	0.866001	4.55*E* − 43	16
EOMES	Pink	0.627819	1.32*E* − 16	0.831907	7.42*E* − 37	15
KLRF1	Pink	0.733463	9.88*E* − 25	0.815474	2.43*E* − 34	15

**Table 2:** Gene expression datasets used in this study.

GEO ID	Samples (CAD : MI)	Platform	Year	Author	Type
*Training set*					
GSE59867	46 : 111	GPL6244	2015	Gora M	Peripheral blood mononuclear cells
GSE62646	14 : 28	GPL6244	2014	Kiliszek M	Peripheral blood mononuclear cells
*Validation set*					
GSE123342	22 : 67	GPL17586	2019	Vanhaverbeke M	Whole blood

## Data Availability

The datasets used and analyzed during the current study are available from the public database Gene Expression Omnibus repository. Accession numbers of the datasets used in current study are GSE59867, GSE62646, and GSE123342 in the Gene Expression Omnibus (https://www.ncbi.nlm.nih.gov/geo).

## Abbreviations

MI: Myocardial infarction
GEO: Gene Expression Omnibus
ROC: Receiver operating characteristic
WGCNA: Weighted gene coexpression network analysis
CAD: Coronary artery disease
GO: Gene Ontology
KEGG: Kyoto Encyclopedia of Genes and Genomes
PPI: Protein-protein interaction
MM: Module membership
GS: Gene significance
NKG7: Natural killer cell granule protein 7
PRF1: Perforin
FASLG: Fas ligand
TBX21: T-box 21 variant
KLRD1: Killer cell lectin-like receptor D1.
